# The Model for End-Stage Liver Disease Score and the Follow-Up Period Can Cause the Shift of Circulating Lymphocyte Subsets in Liver Transplant Recipients

**DOI:** 10.3389/fmed.2021.779443

**Published:** 2022-01-03

**Authors:** Fei Pan, Shuang Cao, Xian-Liang Li, Ya-nan Jia, Ruo-lin Wang, Qiang He, Ji-Qiao Zhu

**Affiliations:** Department of Hepatobiliary and Pancreaticosplenic Surgery, Medical Research Center, Beijing Organ Transplant Center, Beijing Chaoyang Hospital, Capital Medical University, Beijing, China

**Keywords:** liver transplantation, circulating lymphocyte subsets, follow-up period, model for end-stage liver disease score, dynamic changes

## Abstract

Little is known about the shift of lymphocytes under the condition of the model for end-stage liver disease score and the follow-up period. Then, we detected the peripheral blood from liver transplant recipients by flow cytometry and compared the results. The model for end-stage liver disease score affected the percentages of T-cell subsets and B cells during the short-term follow-up period, but failed to influence the lymphocyte subsets during the long-term follow-up period. In contrast, the follow-up period not only affected the absolute counts of T-cell subsets and natural killer (NK) cells in patients with the low model for end-stage liver disease scores, but also influenced the percentages and absolute counts of T-cell subsets in patients with the high model for end-stage liver disease scores. In the two-way ANOVA, we further revealed that the model for end-stage liver disease score was associated with the percentages of T cells and CD4^+^ T cells and the absolute numbers of T-cell subsets and B cells, while the follow-up period was associated with the percentages of T-cell subsets and the absolute numbers of lymphocyte subsets. Therefore, patients with either the low model for end-stage liver disease scores or the long-term follow-up period are in a relatively activated immune condition.

## Introduction

Liver transplantation is a promising procedure for patients with benign and malignant liver diseases. However, these patients are universally plagued by immunosuppression-related complications following liver transplantation, especially acute rejection during the posttransplant period (1–3). Currently, the model for end-stage liver disease (MELD) score has been widely accepted as a fair and objective method for liver transplant allocation, which is based on disease severity (4). The correlation between the preoperative MELD score and the occurrence of acute rejection is uncertain. Jia et al. (5) found that liver transplant recipients (LTR) with rejection had higher MELD scores. Similar results were described in adult-to-adult living donor liver transplantation (6). In contrast, Selzner et al. (7) reported that there were no differences between patients with high or low MELD scores in terms of acute rejection. Actually, different circulating lymphocyte subsets have been repeatedly reported as critical components in acute graft rejection (8–11). Therefore, the correlation could be further investigated between the MELD scores and the lymphocytes. In patients with hepatitis B virus-related acute-on-chronic liver failure, the MELD score was found to be correlated positively with the regulatory T cells to T helper 17 (Th17) ratio at the peak point (12), while negatively with the ratio between circulating CD3^+^ T cells and monocytes (13). These patients also had significantly increased CD4^+^ T cells and decreased lymphocytes CD3^+^ T cells than normal subjects (14). Since studies mainly focus on the relations between T cells and the MELD scores, the impact of the MELD score on lymphocytes remains to be elucidated.

Different follow-up periods might have an association with the occurrence of acute rejection, as most acute rejections reported in LTR occur within the first year, especially the first 6 months (15–17). Zhu et al. noted a group of LTR that showed acute rejection at a mean follow-up of nearly 2 months, whose interferon-γ^+^ (IFN-γ^+^) CD4^+^ T cells and interleukin-2 (IL-2^+^) CD4^+^ T cells and transforming growth factor-β (TGF-β^+^) CD19^+^ B cell and granzyme B^+^ CD19^+^ B cell rose significantly compared with LTR without rejection (9). Boix et al. found that LTR, who rejected the allograft, had a statistically significant higher ratio of CD4^+^ CD154^+^ T cells and CD8^+^ CD154^+^ T cells on the 7th and 15th postoperative days (18). Nevertheless, the recommended tacrolimus trough concentrations used in immunosuppressive schemes taper during the first 6 months (19–21). In contrast, patients, who tend to develop a late opportunistic infection, are found to have lower counts of CD3^+^, CD4^+^, CD8^+^ T cells, and natural killer (NK) cells at the first postoperative month (22, 23). Therefore, in an attempt to better control acute rejection following liver transplantation, it is of great importance to know the rationale of the lymphocyte subset shift over time.

Presently, little is known about the dynamic changes of circulating lymphocyte subsets in LTR with the different MELD scores and the follow-up periods. In the meantime, previous studies mainly focused on a specific cell subset and our understanding of how the circulating lymphocyte subsets as a whole respond to a transplant is lacking. Thus, characterizing the shift of circulating lymphocyte subsets in the MELD score follow-up period co-occurrence would help to understand the postoperative immune status and tailor individualized immunosuppressive therapy. We conducted this study to analyze the effects of the MELD score, the follow-up period, and their possible interaction on the lymphocyte subsets following liver transplantation.

## Materials and Methods

### Study Design

This study was conducted to investigate the MELD score and the follow-up period-related dynamic changes in circulating lymphocyte subsets in LTR, who underwent a single liver transplant and were followed up at the Beijing Chaoyang Hospital between December 2017 and July 2020. To accurately evaluate the effects of the MELD score and the follow-up period on circulating lymphocyte subsets, LTR with concurrent autoimmune disease, HIV, diseases of hematopoietic and lymphoid systems, or any postoperative complications were excluded. We followed the method part of Pan et al. (24) in this study.

### Immunosuppressive Therapy

Immunosuppressive therapy consisted of induction with basiliximab (20 mg on days 0 and 4) and maintenance, which was based on steroids, mycophenolate mofetil, and tacrolimus. Methylprednisolone (500 mg) was intravenously infused during the operation. After surgery, it was given by 240 mg/day and daily reduced by 40 mg till the 6th postoperative day. Then, it was changed to prednisolone (20 mg/day). Prednisolone was gradually withdrawn within 1 month. Sirolimus was used in selected patients with impaired renal function or for its antitumor effects at least 1 month after surgery.

### Cell Preparation and Surface Staining

A sample of 5 ml of whole blood was taken from LTR. The separation of peripheral blood mononuclear cells (PBMCs) was performed *via* Ficoll density gradient centrifugation. After that, PBMCs were resuspended in phosphate-buffered saline (PBS). Then, PBMCs were stained with antibodies at 4°C in the dark for 20 min. The following reagents were obtained from BD Biosciences (Franklin Lakes, New Jersey, USA): fluorescein isothiocyante (FITC)-anti-CD3, CY5.5-anti-CD4, phycoerythrin (PE)-anti-CD19, allophycocyanin (APC)-anti-CD16, and PE-anti-CD56.

### Flow Cytometric Measurement

**Figure 1:**
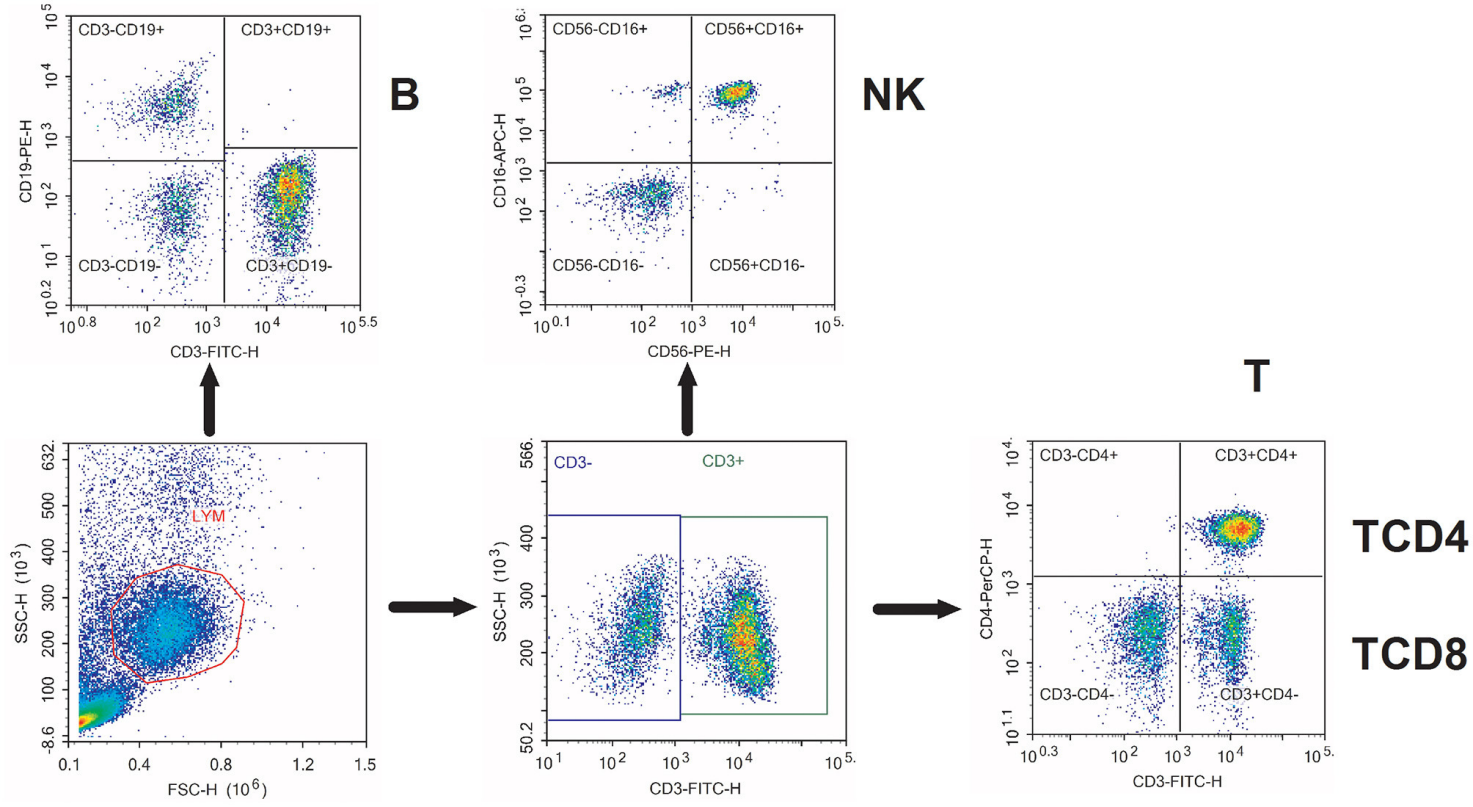
Flow cytometry characterization of circulating lymphocyte subsets. Representative flow cytometry dot plots of the characterization of various circulating lymphocyte subsets. T, CD3^+^ T cells; TCD4, CD3^+^ CD4^+^ T cells; TCD8, CD3^+^ CD8^+^ T cells; B, CD19^+^ B cells; NK, CD56^+^ CD16^+^ natural killer cells.

After surface staining, PBMCs were washed twice with 2 ml PBS and resuspended in 400 μl PBS for flow cytometry analysis. Flow cytometry was conducted on NovoCyte D2060R (ACEA Biosciences Incorporation, San Diego, California, USA). We used the NovoExpress software (San Diego, California, USA) for analysis. The lymphocytes evaluated were T (CD3^+^), TCD4 (CD3^+^ CD4^+^), TCD8 (CD3^+^ CD8^+^), B (CD19^+^), and NK (CD56^+^CD16^+^). Flow cytometry characterization of circulating lymphocyte subsets is given in Figure 1. The lymphocyte subset counts were obtained using the percentages *via* flow cytometry and the absolute numbers of lymphocytes obtained *via* routine blood tests on the same day.

### Statistical Analysis

All the statistical analyses were produced in the SPSS 19.0 computer software (IBM Corporation, Armonk, New York, USA). All the values compared were expressed as mean ± SD. Normal distribution tests were applied by the Kolmogorov–Smirnov test. The chi-square test or Fisher's exact test was employed to compare nominal variables. Student's *t*-test was applied to compare independent samples. The two-way ANOVA was used to test for the MELD score vs. the follow-up period interaction. The results were statistically significant at the 0.05 level. The prism was used for figures.

## Results

### Study Population

As circulating lymphocyte subsets have been reported to be affected under physiological and pathological conditions (25–27), we selected LTR in the absence of any postoperative complications to minimize the potential impact. A total of 66 LTRs with stable liver function were enrolled in this study. There were 7 women and 59 men with a mean age of 52 years (range, 26–73 years). Hepatic carcinoma was pathologically proven in 24 patients, while the rest had hepatitis-related cirrhosis. LTR was divided into four groups depending on the MELD score (low MELD score < 10, high MELD score ≥ 20) and the follow-up period (short-term follow-up < 28 days, long-term follow-up 28 days−3 months) including the LmLf group (low MELD score and long-term follow-up), the HmLf group (high MELD score and long-term follow-up), the LmSf group (low MELD and short-term follow-up), and the HmSf group (high MELD score and short-term follow-up). Characteristics of these patients are given in Table 1.

**Table 1:** Demographic data.

	**LmLf (*n* = 18)**	**HmLf (*n* = 13)**	**LmSf (*n* = 20)**	**HmSf (*n* = 15)**	***P***
Gender (Male)	16	12	18	13	0.830
Age	55.17 ± 7.06	53.62 ± 9.16	52.20 ± 11.65	50.07 ± 15.55	0.221
Hepatic carcinoma	9	4	8	3	0.309
MELD score	3.94 ± 2.45^a^	28.85 ± 4.67^b^	4.45 ± 2.61^c^	29.8 ± 5.94^d^	0.000
Follow-up period	56.78 ± 15.16^a^	56.38 ± 16.52^c^	16.95 ± 5.60^b^	18.07 ± 5.90^d^	0.000

*Lm, low MELD score; Hm, high MELD score; Sf, short-term follow-up; Lf, long-term follow-up; MELD, a model for end-stage liver disease*.

*a vs. b, a vs. d, c vs. b, and c vs. d, p < 0.001*.

### Effect of the MELD Score on Circulating Lymphocyte Subsets

**Figure 2:**
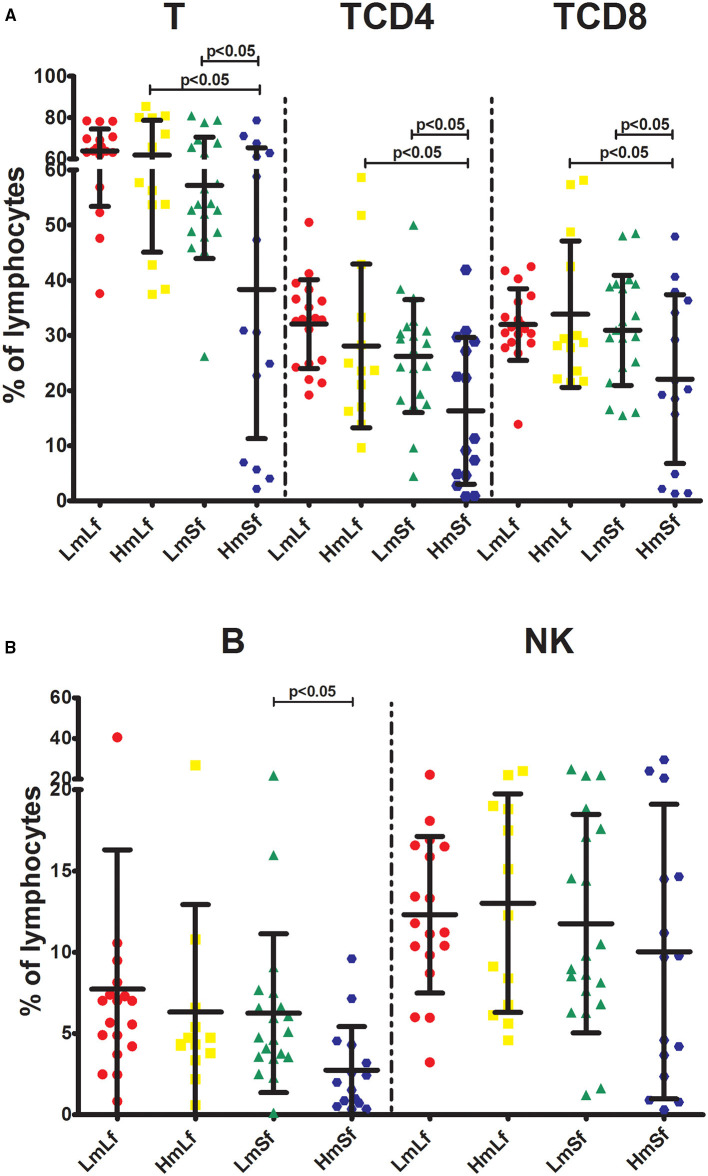
Effects of the MELD score and the follow-up period on the percentages of lymphocyte subpopulations following liver transplantation. Comparison of the percentages of T, TCD4, and TCD8 **(A)**, B and NK **(B)** among LmLf (*n* = 18), HmLf (*n* = 13), LmSf (*n* = 20), and HmSf (*n* = 15). Bars represent mean and SD. MELD, model for end-stage liver disease; T, CD3^+^ T cells; TCD4, CD3^+^ CD4^+^ T cells; TCD8, CD3^+^ CD8^+^ T cells; B, CD19^+^ B cells; NK, CD56^+^ CD16^+^ natural killer cells; LmLf, low MELD score (<10) and long-term follow-up (28 days−3 months); HmSf, high MELD score (≥20) and short-term follow-up (<28 days); HmLf, high MELD score and long-term follow-up; LmSf, low MELD score and short-term follow-up.

**Figure 3:**
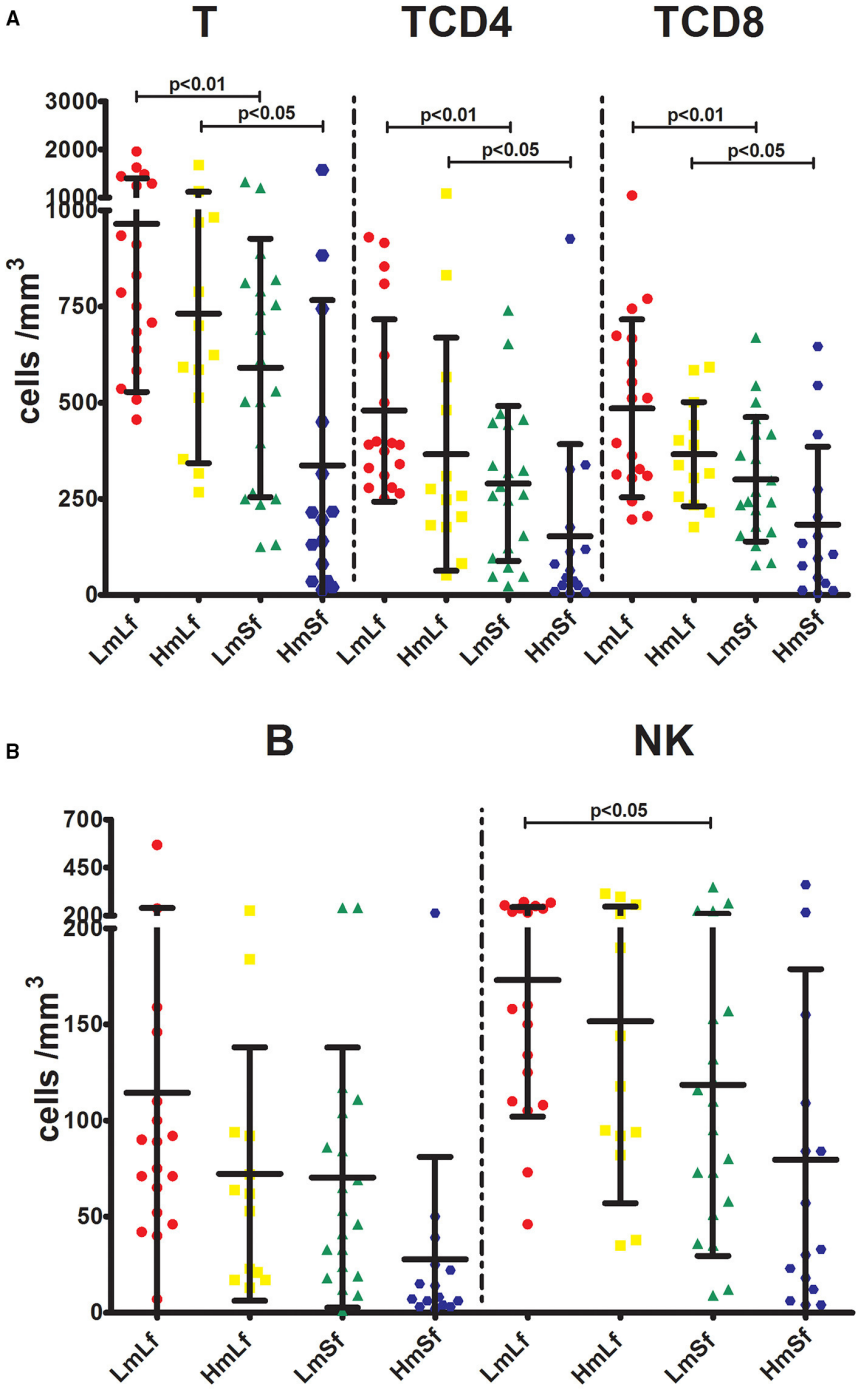
Effects of the MELD score and the follow-up period on the absolute counts of lymphocyte subpopulations following liver transplantation. Comparison of the absolute counts of T, TCD4, and TCD8 **(A)**, and NK **(B)** among LmLf (*n* = 18), HmLf (*n* = 13), LmSf (*n* = 20), and HmSf (*n* = 15). Bars represent mean and SD. MELD, model for end-stage liver disease; T, CD3^+^ T cells; TCD4, CD3^+^ CD4^+^ T cells; TCD8, CD3^+^ CD8^+^ T cells; B, CD19^+^ B cells; NK, CD56^+^ CD16^+^ natural killer cells; LmLf, low MELD score (<10) and long-term follow-up (28 days−3 months); HmSf, high MELD score (≥20) and short-term follow-up (<28 days); HmLf, high MELD score and long-term follow-up; LmSf, low MELD score and short-term follow-up.

First, we wanted to determine whether the MELD score would affect the postoperative circulating lymphocyte subsets. We performed the comparison between the low MELD score (<10) and long-term follow-up (28 days−3 months) (LmLf) group and the high MELD score and long-term follow-up (HmLf) group and the low MELD score and short-term follow-up (LmSf) group and the high MELD score (≥20) and short-term follow-up (<28 days) (HmSf) group. After comparison, we found that there was no statistical difference between the LmLf group and the HmLf group with respect to the percentages (Figure 2 and Table 2) and absolute counts (Figure 3 and Table 3) of T, TCD4, TCD8, B, and NK (*p* > 0.05), suggesting that the MELD score did not affect the lymphocyte subsets during long-term follow-up.

**Table 2:** Effects of the MELD score and the follow-up period on the percentages of lymphocyte subpopulations following liver transplantation.

**Percentages**	**LmLf (*n* = 18)**	**HmLf (*n* = 13)**	**LmSf (*n* = 20)**	**HmSf (*n* = 15)**
T	63.90 ± 10.52	61.89 ± 16.81^*^	57.22 ± 13.29^*^	38.35 ± 27.06
TCD4	32.06 ± 8.06	28.09 ± 14.85^*^	26.24 ± 10.23^*^	16.33 ± 13.30
TCD8	31.97 ± 6.52	33.84 ± 13.28^*^	30.93 ± 9.98^*^	22.09 ± 15.30
B	7.73 ± 8.56	6.32 ± 6.62	6.25 ± 4.88^*^	2.74 ± 2.69
NK	12.31 ± 4.82	13.02 ± 6.73	11.76 ± 6.73	10.03 ± 9.06

*MELD, model for end-stage liver disease; T, CD3^+^ T cells; TCD4, CD3^+^ CD4^+^ T cells; TCD8, CD3^+^ CD8^+^ T cells; B, CD19^+^ B cells; NK, CD56^+^ CD16^+^ natural killer cells; Lm, low MELD score; Hm, high MELD score; Sf, short-term follow-up; Lf, long-term follow-up*.

*HmLf vs. HmSf, LmSf vs. HmSf*,

* *p < 0.05*.

**Table 3:** Effects of the MELD score and the follow-up period on the absolute counts of lymphocyte subpopulations following liver transplantation.

**Absolute counts**	**LmLf (*n* = 18)**	**HmLf (*n* = 13)**	**LmSf (*n* = 20)**	**HmSf (*n* = 15)**
T	964.72 ± 437.14^**^	731.62 ± 388.89^*^	590.60 ± 335.74	336.40 ± 429.80
TCD4	479.61 ± 236.73^*^	365.69 ± 302.93^*^	289.95 ± 201.47	153.00 ± 239.11
TCD8	485.22 ± 231.35^**^	365.92 ± 135.42^*^	300.65 ± 161.45	183.47 ± 201.86
B	114.44 ± 124.70	72.15 ± 65.96	70.25 ± 67.68	27.80 ± 53.15
NK	173.11 ± 71.15^*^	151.54 ± 94.55	118.60 ± 89.08	79.67 ± 99.06

*MELD, model for end-stage liver disease; T, CD3^+^ T cells; TCD4, CD3^+^ CD4^+^ T cells; TCD8, CD3^+^ CD8^+^ T cells; B, CD19^+^ B cells; NK, CD56^+^ CD16^+^ natural killer cells; Lm, low MELD score; Hm, high MELD score; Sf, short-term follow-up; Lf, long-term follow-up*.

*HmLf vs. HmSf, LmSf vs. HmSf*,

* *p < 0.05*,

** *p < 0.01*.

In contrast, we observed higher percentages of T, TCD4, TCD8, and B (Figure 2 and Table 2) in the LmSf group (*p* < 0.05), but the similar percentage of NK (Figure 2 and Table 2) and absolute counts (Figure 3 and Table 3) of T, TCD4, TCD8, B, and NK (*p* > 0.05) when the LmSf group was compared with the HmSf group. The results mean that the MELD score could affect the lymphocyte subsets during short-term follow-up.

### Effect of the Follow-Up Period on Circulating Lymphocyte Subsets

Next, we checked whether the follow-up period played an important role in the shift of circulating lymphocyte subsets. We compared the LmLf group with the LmSf group and subsequently found that the comparison failed to reach significance although the percentages (Figure 2 and Table 2) of T, TCD4, TCD8, B, and NK were a little higher in the LmLf group (*p* > 0.05). However, the absolute counts (Figure 3 and Table 3) of T-cell subsets and NK cells rather than B cells were significantly different between the groups (*p* < 0.05). These results showed that T-cell subsets and NK cells from patients with mild liver disease proliferated significantly over the follow-up period.

Surprisingly, the HmSf group presented lower percentages (Figure 2 and Table 2) and lower absolute numbers (Figure 3 and Table 3) of T-cell subsets (*p* < 0.05), but no significant differences in percentages (Figure 2 and Table 2) and absolute numbers (Figure 3 and Table 3) of B and NK (*p* > 0.05) were observed when compared with the HmLf group. These data reflected that T-cell subsets from patients with severe liver disease also proliferated significantly over the follow-up period.

### Interaction of the MELD Score and the Follow-Up Period on Circulating Lymphocyte Subsets

From the above analysis, we noticed that the MELD score and the follow-up period could affect the circulating lymphocyte subsets. Therefore, we, then investigated the combined effect of the MELD score and the follow-up period on circulating lymphocyte subsets. To strengthen the analysis of the combined effects and to test their possible interaction, we performed the two-way ANOVA with four groups in a two-by-two factorial design. The independent variables were the MELD score (high vs. low) and the follow-up period (short term vs. long term).

After comparison, we found that there was no synergetic effect on the percentages or absolute counts of lymphocyte subsets (Table 4). In this analysis, the MELD score influenced the percentages of T and TCD4 and the absolute numbers of T, TCD4, TCD8, and B (*p* < 0.05), while the follow-up period had an impact on the percentages of T, TCD4, and TCD8 and the absolute numbers of T, TCD4, TCD8, B, and NK (*p* < 0.05). The results found in the two-way ANOVA are a little different from the above findings.

**Table 4:** The two-way ANOVA for the MELD score, the follow-up period, and the MELD score × the follow-up period interaction.

**Lymphocyte subsets**	**Percentages**			**Absolute counts**		
	**MELD score**	**Follow-up period**	**MELD score x follow-up period**	**MELD score**	**Follow-up period**	**MELD score x follow-up period**
T	0.020	0.001	0.057	0.017	0.000	0.916
TCD4	0.019	0.003	0.305	0.042	0.001	0.849
TCD8	0.224	0.028	0.063	0.014	0.000	0.982
B	0.112	0.103	0.496	0.049	0.040	0.997
NK	0.770	0.309	0.481	0.174	0.006	0.694

*MELD, model for end-stage liver disease; T, CD3^+^ T cells; TCD4, CD3^+^ CD4^+^ T cells; TCD8, CD3^+^ CD8^+^ T cells; B, CD19^+^ B cells; NK, CD56^+^ CD16^+^ natural killer cells*.

## Discussion

In this study, data from LTR with the different MELD scores at the different follow-up periods were collected and analyzed to determine the function of the two factors on the shift of circulating lymphocyte subsets. We found that the MELD score and the follow-up period did not have a synergetic effect on the percentages or absolute counts of lymphocyte subsets. The MELD score affected the percentages of T and TCD4 and the absolute numbers of T-cell subsets and B cells. The follow-up period was in relation to the percentages of T-cell subsets and the absolute numbers of lymphocyte subsets.

Currently, extensive reports have described the shift of a specific cell subpopulation in various diseases (28–30). Nevertheless, there are only limited studies on the changes in lymphocyte subsets under the condition of the MELD score following the liver transplantation. It was found that patients with acute-on-chronic liver failure had high Th17 frequency since the onset point (12) and the decreased T-cell repertoire (31). Freitas et al. reported that patients with benign renal diseases had lower absolute counts of T-cell subsets and B cells when compared with healthy controls (32). These studies reflect that patients with high MELD scores have suppressed immunity. In this study, we also confirmed that LTR with the high MELD scores had lower absolute counts of T-cell subsets and B cells. Since liver failure can lead to a significant decrease in the percentage of CD4^+^ T cells (33), we detected a rather lower percentage of CD4^+^ T cells in LTR with the high MELD scores. In addition, Tanimine et al. (34) demonstrated that chronic liver disease could also impair the potential of intrahepatic NK cells, which is not in agreement with our finding. Of note, dysfunction of NK cells rather than the percentage or the cell number is favored by poor clinical outcomes in liver cancer (35, 36). In this study, circulating NK cells were detected and LTR with both the malignant and benign liver diseases was enrolled. Therefore, this might, at least in part explain that the MELD score failed to affect the percentages and absolute counts of NK cells.

At present, the impact of the follow-up period on circulating lymphocyte subsets following liver transplantation is unclear. Zhuang et al. detected that the cell counts of T, TCD4, TCD8, B, and NK dropped profoundly shortly after stereotactic body radiation therapy and gradually recovered 2 months later (37). Similarly, we observed that the absolute numbers of lymphocyte subsets were reduced in LTR with short-term follow-up. Moreover, we found that the follow-up period affected the percentages of T-cell subsets instead of B cells and NK cells. At our center, basiliximab was used for induction and steroids and tacrolimus were used for maintenance. After the administration of prednisone and tacrolimus, there was a profound lymphocytopenia, a selective decrease in T cells (38–40). Nevertheless, there was no relation between prednisone or tacrolimus trough level and B cells, as mycophenolate mofetil has an impact on the suppression of B-cell functions (41, 42). Therefore, the percentages of T-cell subsets are relatively lower during short-term follow-up. With the rapid recovery of circulating lymphocyte subsets over time, there was a significant rise in the percentages of T-cell subsets.

From the above analysis, we know both the MELD score and the follow-up period can affect the circulating lymphocyte subsets, which show the clinical role in regulating the dose of immunosuppressive drugs. Since the MELD score is calculated based on the parameters of liver function and renal function, LTR with the high MELD scores usually has deteriorated renal function. Additionally, calcineurin inhibitors can further worsen renal function. Exposure to high serum levels of calcineurin inhibitors can result in infection, although the occurrence rate of acute rejection is scarce. Therefore, it is possible to employ a lower dose of calcineurin inhibitors in patients with high MELD scores following the liver transplantation. On the other hand, with the patients' recovery over time, their immunity improves, which makes the patients vulnerable to acute rejection. Hence, the dose of calcineurin inhibitors should increase accordingly. In contrast, current guidelines recommend that tacrolimus trough concentrations are high shortly after surgery, while it is maintained at a relatively low level over time (19–21), which might account for the higher occurrence rate of acute rejection within the first 6 months (15, 16). Taken together, based on the individual immune status instead of empiric therapy, the tailored treatment for each recipient is more favored.

The main limitation of this study is the relatively small number of patients enrolled that may limit the accuracy of our assessment. However, to make the data among different groups comparable, we excluded LTR with any complications. Second, the results represented the experience of a single center in selected patients. Future studies, preferably larger patient cohorts from multicenters, are needed to further confirm our preliminary outcomes.

## Conclusion

Liver transplant recipients with either the low MELD scores or the long-term follow-up period are in a relatively activated condition and should be exposed to higher levels of immunosuppressive drugs to prevent immunosuppression-related complications.

## Data Availability Statement

The original contributions presented in the study are included in the article/supplementary material, further inquiries can be directed to the corresponding authors.

## Ethics Statement

The studies involving human participants were reviewed and approved by Institutional Review Board of Beijing Chaoyang Hospital. The patients/participants provided their written informed consent to participate in this study.

## Author Contributions

J-QZ and QH contributed conception and design of the study. FP, R-lW, and SC organized the database. X-LL and Y-nJ performed the statistical analysis. FP and SC wrote the first draft of the manuscript. The rest wrote sections of the manuscript. All the authors contributed to manuscript revision, read, and approved the submitted version. They were accountable for all the aspects of the work in ensuring that questions related to the accuracy or integrity of any part of the work are appropriately investigated and resolved.

## Funding

This study is supported by the Open Project of Beijing Key Laboratory of Tolerance Induction and Organ Protection in Transplantation (2017YZNS01) and the National Natural Science Foundation of China (81601392).

## Conflict of Interest

The authors declare that the research was conducted in the absence of any commercial or financial relationships that could be construed as a potential conflict of interest.

## Publisher's Note

All claims expressed in this article are solely those of the authors and do not necessarily represent those of their affiliated organizations, or those of the publisher, the editors and the reviewers. Any product that may be evaluated in this article, or claim that may be made by its manufacturer, is not guaranteed or endorsed by the publisher.
